# Detection of Carbapenemase-Producing Enterobacteriaceae in the Baltic Countries and St. Petersburg Area

**DOI:** 10.1155/2014/548960

**Published:** 2014-03-04

**Authors:** Anastasia Pavelkovich, Arta Balode, Petra Edquist, Svetlana Egorova, Marina Ivanova, Lidia Kaftyreva, Irina Konovalenko, Siiri Kõljalg, Jana Lillo, Lidia Lipskaya, Jolanta Miciuleviciene, Kristiine Pai, Kristel Parv, Katri Pärna, Tiiu Rööp, Epp Sepp, Jelena Štšepetova, Paul Naaber

**Affiliations:** ^1^Department of Microbiology, University of Tartu, Ravila 19, 50411 Tartu, Estonia; ^2^East-Tallinn Central Hospital, Ravi 18, 10138 Tallinn, Estonia; ^3^Rīga Stradiņš University, 16 Dzirciema Street, Rīga, LV-1007, Latvia; ^4^Smittskyddsinstitutet, Folkhälsomyndigheten, 171 82 Solna, Sweden; ^5^Institut Pasteur in Saint Petersburg, Ul Mira 14, Saint Petersburg 197101, Russia; ^6^St. Petersburg Hospital No. 31, Pr. Dinamo 3, Saint Petersburg 197110, Russia; ^7^St. Petersburg Hospital No. 40, Ul Borisova 9, Sestroretsk, Saint Petersburg 197706, Russia; ^8^Vilnius City Clinical Hospital, Antakalnio Street 57, LT-10007 Vilnius, Lithuania; ^9^Quattromed HTI Laboratories, Väike-Paala 1, 11415 Tallinn, Estonia

## Abstract

The spread of carbapenemase-producing Enterobacteriaceae is a global problem; however, no exact data on the epidemiology of carbapenemase in the Baltic countries and St. Petersburg area is available. We aimed to evaluate the epidemiology of carbapenemase-producing *Escherichia coli* and *Klebsiella pneumoniae* in the Baltic States and St. Petersburg, Russia, and to compare the different methods for carbapenemase detection. From January to May 2012, all *K. pneumoniae* (n = 1983) and *E. coli* (n = 7774) clinical isolates from 20 institutions in Estonia, Latvia, Lithuania, and St. Petersburg, Russia were screened for carbapenem susceptibility. The IMP, VIM, GIM, NDM, KPC, and OXA-48 genes were detected using real-time PCR and the ability to hydrolyze ertapenem was determined using MALDI-TOF MS. Seventy-seven strains were found to be carbapenem nonsusceptible. From these, 15 *K. pneumoniae* strains hydrolyzed ertapenem and carried the *bla*_NDM_ gene. All of these strains carried integron 1 and most carried integron 3 as well as genes of the CTX-M-1 group. No carbapenemase-producing *E. coli* or *K. pneumoniae* strains were found in Estonia, Latvia, or Lithuania; however, NDM-positive *K. pneumoniae* was present in the hospital in St. Petersburg, Russia. A MALDI-TOF MS-based assay is a suitable and cost-effective method for the initial confirmation of carbapenemase production.

## 1. Introduction

Antibiotic resistance remains a major global public health problem that leads to increasing healthcare costs, extra length of hospital stay, and treatment failures [1]. The recent European Antimicrobial Resistance Surveillance Network (EARS-Net) report indicates that although the occurrence of antibiotic resistance in gram-positive pathogens appears to be stabilizing or even decreasing in some countries Europe-wide increase in antimicrobial resistance in gram-negative pathogens, such as *Escherichia coli* and *Klebsiella pneumoniae*, is occurring [1].

An emerging problem is the spread and increasing prevalence of carbapenemase-producing gram-negative bacteria. For example, outbreaks of *Klebsiella pneumoniae* carbapenemase (KPC)-positive *K. pneumoniae* occurred in the USA in 2001 and subsequently spread throughout the world; New Delhi metallo-*β*-lactamase (NDM-1)-positive *K. pneumoniae* was imported from India and spread to the United Kingdom in 2010 [2]. As high as a 50% prevalence of metallo-*β*-lactamases were reported in *K. pneumoniae* blood isolates in Greece [3].

In a recent European study, most participants declared sporadic cases or outbreaks of carbapenemase-producing Enterobacteriaceae [4]. Although carbapenem nonsusceptible* E. coli* and *K. pneumoniae* cases have also been reported in the Baltic states, no exact epidemiological data are available, and these cases were not confirmed using molecular methods.

The detection of carbapenemases in clinical microbiology labs is challenging [5] because phenotypic tests are time consuming and difficult to interpret and the molecular methods are not available in routine diagnostic labs in our region. Additionally, the increasing number of new carbapenemases makes molecular tests unsuitable for the initial detection of carbapenemase production. Recently a MALDI-TOF MS (matrix-assisted laser desorption ionization-time of flight mass spectrometry) assay has been described as a potentially useful tool for the detection of *β*-lactamase activity [6, 7]. This technique is based on the detection of *β*-lactams degradation products. However, this method has not yet been introduced in routine diagnostics or validated in different geographical regions.

The aim of our study was to evaluate the epidemiology of carbapenemases in the Baltic countries and St. Petersburg area by applying different mass-spectrometry and molecular-based detection methods.

## 2. Materials and Methods

### 2.1. Collection of Strains

A total of 20 institutions from the following Baltic countries and St. Petersburg area participated in our study: Estonia (*n* = 5), Lithuania (*n* = 3), Latvia (*n* = 4), and the Saint-Petersburg region in Russia (*n* = 8). From January 1 to May 31, 2012, all isolated *E. coli* and *K. pneumoniae* nonduplicated strains were screened for carbapenem nonsusceptibility according to European Committee of Antimicrobial Susceptibility Testing (EUCAST 2.0; applied in Baltic states) or Clinical and Laboratory Standard Institute (CLSI 2011; applied in Russia) standards in the participating labs. Carbapenem nonsusceptibility to ertapenem and/or meropenem was detected using disc-diffusion or broth dilution methods. Strains from all clinically relevant materials that were routinely sent to these microbiology labs were included. Environmental and surveillance samples were not included in our study. In total, 9757 strains were isolated and screened for carbapenem nonsusceptibility and included *E. coli* from hospitalized patients (*n* = 4799) and outpatients (*n* = 2975) and *K. pneumoniae* from hospitalized patients (*n* = 1631) and outpatients (*n* = 352). All screening positive strains were sent to and further investigated in the Estonian central lab.

### 2.2. Strains Data

Institution (numbers of beds, admissions, and patient days), strain-related (sampling date, origin of isolation/material, and resistance pattern detected in the local labs), and patient-related data (patient age, outpatient or hospitalized patient, and department type) were also collected for the carbapenem nonsusceptible strains.

### 2.3. Phenotypic Description of Strains

Species identification of these strains was confirmed in the central lab using MALDI-TOF MS (Maldi Biotyper, Bruker Daltonics GmbH, Germany). Carbapenem nonsusceptibility was confirmed using the agar-gradient method with ertapenem, meropenem, and imipenem strips (Liofilchem, Italy) on Muller-Hinton agar (Oxoid Limited, UK) according to the manufacturer's recommendations and was interpreted according to EUCAST standards (http://www.eucast.org/).

### 2.4. Mass-Spectrometry-Based Confirmation of Carbapenemase Production

Carbapenem nonsusceptible strains were analyzed using a mass-spectrometry-based semi quantitative resistance assay method that was described previously by Burckhardt and Sparbier [8, 9] and modified according to Dr. Katrin Sparbier's recommendations (personal communication).

Bacterial strains were grown for 18 to 24 h on 5% sheep blood agar plates (Microlabor, Estonia) at 36°C. The cells were then resuspended in thirty microliters of 0.45% NaCl solution with or without 1 g/litre ertapenem (MSD, USA) in 1.5-mL Eppendorf tubes and the amount of bacteria filled a 1-*μ*L-inoculation loop (corresponding to 3–6 × 10^8^ cells, 1 mL of a McFarland 1-2). The suspension was incubated for 2 h at 37°C under agitation and then centrifuged for 2 min at 13.000 rpm at 20°C. One microliter of the cleared supernatant was added to each target spot and dried at room temperature, and 1 *μ*L of matrix (HCCA [*α*-cyano-4-hydroxycinnamic acid], high-pressure liquid chromatography [HPLC] grade; Fluka, Germany) was added to each target spot.

MALDI-TOF MS analysis was performed using a Microflex LT instrument (Bruker Daltonics GmbH, Germany) and 96-spot, polished-steel targets. The specifically modified protocol was used (0 to 1 000 Da, 12 to 17% laser intensity). For one spectrum, approximately 200 to 300 shots were summed and the HCCA peak at 379 Da was used for calibration. The mass spectrum between 438 and 530 Da was analyzed using 3.0 software flexAnalysis (Bruker Daltonics GmbH, Germany). Carbapenem nonsusceptible strains were analyzed in six reaction sets and each set contained positive (carbapenemase-producing strain) and negative controls (strain incapable of carbapenemase production), and during each reaction set, the characteristic mass spectrum of pure ertapenem was measured.

An automated evaluation algorithm for the MALDI-TOF MS-based functional *β*-lactamase assay (MSBL assay) was implemented using Bruker Daltonics software prototype (which is underdevelopment and not yet commercially available) that was employed in cooperation with Sparbier et al. [10]. This software automatically calculates the logarithm of the sum ratio of the intensity of all peaks that corresponded to the hydrolyzed form and the sum of the intensities of all peaks that corresponded to the non-hydrolyzed form of the antibiotic (logRQ). The results were displayed in a box plot diagram [10]. This automated evaluation was used as a control method for the visual analysis of the spectra.

### 2.5. Molecular Characterization of Strains

Total bacteria DNA from the clinical samples was extracted using the QIAamp DNA Mini Kit (Qiagen, Germany). Plasmid DNA was extracted using the NucleoSpin Plasmid Quick Pure kit (Macherey-Nagel, France).

Genes encoding IMP, VIM, GIM, NDM, OXA-48, and KPC-group carbapenemases were detected using a multiplex real-time PCR reaction that was based on the protocols published previously by Mendes et al. [11], Poirel et al. [12], Samuelsen et al. [13], and Chen et al. [14]. Amplification was performed in a 20-*μ*L mixture that contained 10 *μ*L of a Power SYBR Green PCR Master Mix (Applied Biosystems, UK) and six pairs of primers (concentration rate is 10 *μ*M) in 3 different PCRs (Table 1(a)) using Applied Biosystems StepOnePlus Real-Time PCR system (Life Technologies, USA). The PCR conditions were as follows: 1 cycle at 50°C for 2 min, initial denaturation at 94°C for 5 min; 35 cycles of 94°C for 20 s, 53°C for 45 s for the *bla*_IMP_, *bla*_VIM_, and *bla*_GIM_ genes, or 60°C for 45 s for the *bla*_OXA-48_, *bla*_NDM_, and *bla*_KPC_ genes, and 72°C for 30 s; and a melt curve step at 95°C for 15 s, 60°C for 1 min, and 95°C for 15 s.

The presence of different *bla*_CTX-M_ subgroups was determined using a multiplex real-time PCR protocol [15] that displayed the group designation (CTX-M-1/-2/-9 and 8/25) of the *bla*_CTX-M_-positive isolates. The method used one primer pair that was specific for all four groups. Four TaqMan probes were included in the reactions and bound to PCR products according to the CTX-M subgroup.

To detect integrons, real-time PCR was performed as previously described [16] with a reaction mixture of 25 *μ*L that contained 5 *μ*L of DNA template, 12.5 *μ*L of TaqMan universal master mix (Applied Biosystem, USA), 6 mM MgCl_2_, 0.4 *μ*M of each primer (*int1*-(LC1-LC5), *int2*-(LC2-LC3), and *int3*-(LC1-LC2)), and 0.2 *μ*M of each probe (*int1*, *int2*, and *int3*; Table 1(b)). The programme was as follows: 10 min initial step at 95°C, followed by 45 cycles at 95°C for 30 sec and 60°C for 1 min, and it was performed using a 7500 real-time PCR detection system (Applied-Biosystem, USA) and optical grade, 96-well plates. Assays were performed in triplicate, and a negative control was included on each plate.

### 2.6. Quality Control Strains

In the carbapenemases detection studies (MALDI-TOF based assay and real-time PCR) the following control strains were used: Swedish Institute for Communicable Disease Control (Sweden) carbapenemases detection control set including IMP-positive *Pseudomonas aeruginosa* Ps540, VIM-positive *K. pneumoniae* CCUG58547, GIM-positive *P. aeruginosa*, NDM-1-positive *K. pneumoniae* K275, OXA-48-positive *K. pneumoniae* Oxa241, KPC-positive *K. pneumoniae* K271, and MBL-positive *Klebsiella pneumoniae* KSKS2823 and EuSCAPE (European survey on carbapenemase-producing Enterobacteriaceae) project quality control set (provided by NEQAS) including KPC-positive *K. pneumoniae* 1940, KPC-2-positive *K. pneumoniae* 1944, KPC-3-positive *K. pneumoniae* 1942, OXA-48-positive *K. pneumoniae* 1943, VIM-1-positive *K. pneumoniae* 1945, NDM-1-positive *K. pneumoniae* strains 1946 and 1948, IMP-1-positive *K. pneumoniae* 1947, and NDM-1-positive *Enterobacter aerogenes* 1941.

In CTX-M detection studies we used *E. coli* CCUG55971 (CTX-M-1 group), *E. coli* CCUG55972 (CTX-M-2), *E. coli* CCUG55970 (CTX-M-9), and *E. aerogenes *isolate Rio3 (CTX-M-8/25) as positive controls. These strains were provided from Swedish Institute for Communicable Disease Control.

For integron detections the following strains were used as positive templates: *E. coli*/pBad::*int1*; *E. coli*/pGEMTeasy::*int2*; and *E. coli*/pBad::*int3* provided from the Department of Bacteriology of University of Limoges (France).

For negative controls wild-type *E. coli* and *K. pneumoniae* clinical strains were used.

## 3. Results

**Figure 1:**
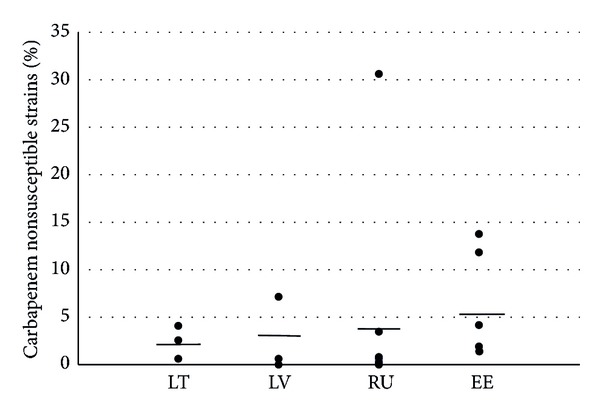
Percentages of carbapenem nonsusceptible *Klebsiella pneumoniae* strains in particular hospitals (dots) and the country averages (lines). LT: Lithuania, LV: Latvia, RU: Russia, St. Petersburg, and EE: Estonia.

According to initial screening and identification confirmation, 80 isolates appeared to be nonsusceptible to carbapenem. After minimal inhibitory concentration (MIC) confirmation using the agar-gradient method, 77 strains had MIC values above the epidemiological cut-off according to the EUCAST standard for any tested carbapenem (nonwild type strains), including 73 *K. pneumoniae* and 4 *E. coli* strains. The prevalence of the carbapenem nonsusceptible strains in the institutions of the participating countries is shown in Figure 1.

### 3.1. Carbapenemase Production Confirmation by MALDI-TOF MS

**Figure 2:**
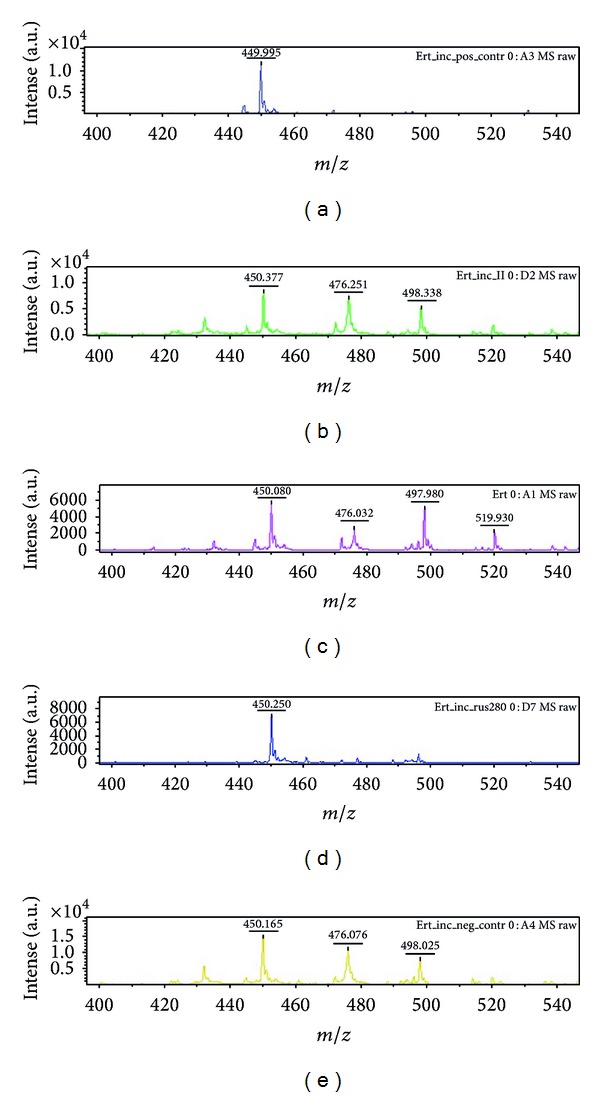
MALDI-TOF MS spectrum showing ertapenem and its degradation products. Spectrum related to (a) positive control (*Klebsiella pneumoniae* metallo-*β*-lactamase positive): hydrolyzed decarboxylated product of ertapenem (450.5 Da); (b) incubated ertapenem solution: hydrolyzed decarboxylated product of ertapenem (450.5 Da), ertapenem molecule (476.5 Da), and ertapenem sodium adduct (498.5 Da); (c) pure ertapenem solution: hydrolyzed decarboxylated product of ertapenem (450.5 Da), ertapenem molecule (476.5 Da), ertapenem sodium adduct (498.5 Da), and ertapenem disodium adduct (520.5 Da); (d) New Delhi Metallo-*β*-lactamase-positive strain (*Klebsiella pneumoniae*): hydrolyzed decarboxylated product of ertapenem (450.5 Da); (e) negative control (noncarbapenemase-producing *Klebsiella pneumoniae*): hydrolyzed decarboxylated product of ertapenem (450.5 Da), ertapenem molecule (476.5 Da), and ertapenem sodium adduct (498.5 Da).

**Figure 3:**
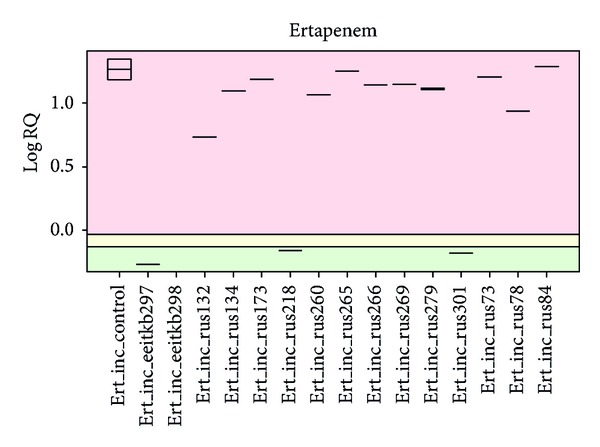
Evaluation of the MALDI-TOF MS spectra of 16 different *Klebsiella pneumoniae* strains using the new software prototype. Differences in the hydrolysis rates of the different strains can easily be detected: green area: no hydrolysis; yellow area: intermediate hydrolysis; red area: hydrolysis.

Of the 77 phenotypically carbapenem nonsusceptible strains, 15 were able to hydrolyze ertapenem, as detected by the MSBL assay. The spectra of all carbapenemase-producing strains showed the hydrolyzed decarboxylated product of ertapenem (450.5 Da), which corresponded to high-strength carbapenemase (Figure 2). The visual- and software-based evaluations of the results gave the same results. An example of the ertapenem hydrolyzation spectra visualised with software prototype is presented in Figure 3.

### 3.2. Molecular Detection of Carbapenemase-Encoding Genes

When analysing these 77 carbapenem nonsusceptible isolates for carbapenemase-encoding genes, we found 15 isolates that were positive for the *bla*_NDM_ gene. All of the other 62 isolates were negative for the *bla*_IMP_, *bla*_VIM_, *bla*_GIM_, *bla*_OXA-48_, *bla*_NDM_, or *bla*_KPC_ genes. The 15 isolates with the NDM gene were also found to hydrolyze ertapenem by the MSBL assay.

### 3.3. Characterization of Strains

All carbapenemase producers were identified as *K. pneumoniae *and were isolated from one St. Petersburg (Russia) hospital (Table 2). Most of these strains were isolated from intensive care patients and were isolated from different clinical materials. All of these strains carried integron 1, and most carried integron 3 and the CTX-M-1 group of genes. All strains were resistant to all of the tested carbapenems, fluoroquinolones, and aminoglycosides. When comparing the minimal inhibitory concentration ranges of the carbapenemase producers and nonproducers, we found that the producers had imipenem MICs ≥8 *μ*g/mL and nonproducers had MICs ≤4 *μ*g/mL. For the other carbapenems the ranges overlapped.

## 4. Discussion

This was the first study to investigate carbapenem resistance in the Baltic countries and St. Petersburg area that included patients and sample types from the multiple largest medical institutions in Estonia, Latvia, Lithuania, and St. Petersburg and to apply molecular methods for characterization of strains. We found high variation in carbapenem nonsusceptibility (MICs above the epidemiological cut-off values) between different institutions within particular countries (highest in Russia: from 0% to 30%). However, variations between the country averages were not so remarkable. Although we found carbapenem nonsusceptible strains in the Baltic states (Estonia, Latvia, and Lithuania), as was described in previous EARS-Net reports, none of these were actually carbapenemase producers. Therefore, after screening nearly 10000 *E. coli* and *K. pneumoniae* strains, we conclude that carbapenemases are not yet a problem in Baltic countries. In these carbapenem nonsusceptible strains, some other mechanisms, for example, production of CTX-M as well as acquired cephalosporinases along with porin loss or changes in efflux, are suspected. In these cases, carbapenem resistance was usually low (relatively low MICs) and thus infections could be treated with higher doses of carbapenems. These strains appear to exhibit low spreading potential. On the contrary, carbapenemase producers are associated with therapy failures, outbreaks, and increased mortality [17]. However, our study revealed high prevalence of NDM-type carbapenemase-producing *K. pneumoniae* from one St. Petersburg hospital. Infection control and antimicrobial resistance surveillance institutions of neighboring Baltic States should be alerted when spreading of this strain occurs.

In our study, we also evaluated different methods, such as MALDI-TOF, MICs of different carbapenems, and real-time PCR, for the detection of carbapenemases in *E. coli *and *K. pneumoniae* isolates. In this set of strains, we found good correlation between functional assay, such as the MALDI-TOF assay and molecular detections of specific carbapenemase genes. Thus, from a sensitivity and specificity point of view, we found these methods to be equal. However, all of these methods have some advantages and disadvantages, as discussed below. The limitation of our study for evaluating the different methods was the epidemiological situation in the studied region, where only one type (NDM) of carbapenemase was present.

Whereas the molecular methods and the usage of carbapenems and the combinations of carbapenems and carbapenemase inhibitors have been available for years for carbapenemase detection, the MALDI-TOF assay is a novel method that is not fully standardized and has not been tested for different strains sets and epidemiological situations. However, carbapenem resistance detection using MALDI-TOF MS has many advantages when compared to synergy/inhibition-based phenotypic methods or PCR. First, depending on the type of carbapenemase, the results can be available as soon as 2-3 h after the start of incubation. This is especially useful in an outbreak situation when the carbapenemase has already been identified. The principle of degradation-product monitoring is universal for the detection of other enzymatic resistance mechanisms, such as extended spectrum *β*-lactamases (ESBL). Second, this method is comparatively easy to perform [8]. Third, the cost per determination is relatively low and is less than one EUR per reaction [8]. The only problem may be the cost of the equipment. However, MALDI-TOF MS has become increasingly used in routine diagnostic labs over recent years for the identification of pathogens.

Unfortunately, a semiquantitative assay does not allow for specification of the enzyme. Currently, a software tool supporting an automated evaluation is under development which will be able to calculate the hydrolysis capacity (Bruker Daltonics GmbH, Germany) [18]. A prototype of this software was also used in our study to compare it with the manual method, and it was easy to use and gave reliable results with our strain set.

PCR is the fastest way to determine which family of *β*-lactamase is present. However, PCR techniques are not used in the majority of routine laboratories because of high costs, absence of real-time equipment, and qualified personnel. In addition, this method can detect only previously described enzymes. Mutations in a carbapenemase-encoding gene could lead to negative results.

We found that of the carbapenem nonsusceptible strains, most carbapenemase producers and nonproducers carried the CTX-M 1 group of ESBL genes and class 1 integrons. Although the class 3 integron was also found in carbapenemase nonproducers, it was more common in NDM-positive *K. pneumoniae* strains. In previous studies, a high prevalence of class 1 integrons in *K. pneumoniae* and its relation with NDM genes have been reported [19–21]. However, relations between carbapenem nonsusceptibility (carbapenemase related or nonrelated) and the presence of class 3 integrons have not yet been described and require further investigation.

In conclusion, carbapenemase-producing *E. coli* or *K. pneumoniae* were not found in Baltic countries, but NDM-positive *K. pneumoniae* was present in St. Petersburg. In our current epidemiological and economic situation, the MALDI-TOF assay seems to be suitable and cost-effective method for the initial confirmation of carbapenemase production.

## Figures and Tables

**Table:** (a)

Reaction number	Primers	Oligonucleotide sequence (5′-3′)	Volume for reaction
PCR Mix 1	IMP (forward)	GAATAGRRTGGCTTAAYTCTC	2.5 *µ*L
	IMP (reverse)	CCAAACYACTASGTTATC	2.5 *µ*L
	VIM (forward)	GTTTGGTCGCATATCGCAAC	0.25 *µ*L
	VIM (reverse)	AATGCGCAGCACCAGGATAG	0.25 *µ*L
	GIM (forward)	TCAATTAGCTCTTGGGCTGAC	0.25 *µ*L
	GIM (reverse)	CGGAACGACCATTTGAATGG	0.25 *µ*L

PCR Mix 2	NDM (forward)	CAATATTATGCACCCGGTCG	0.5 *µ*L
	NDM (reverse)	ATCATGCTGGCCTTGGGGAA	0.5 *µ*L
	OXA 48 (forward)	GCGTGGTTAAGGATGAACAC	0.5 *µ*L
	OXA 48 (reverse)	CATCAAGTTCAACCCAACCG	0.5 *µ*L

PCR Mix 3	KPC (forward)	GGCAGTCGGAGACAAAACC	0.5 *µ*L
	KPC (reverse)	CCCTCGAGCGCGAGTCTA	0.5 *µ*L

**Table:** (b)

Primers/probes	Oligonucleotide sequence (5′-3′)	Location
*intI*1-LC1	GCCTTGATGTTACCCGAGAG	*intI*1
*intI*1-LC5	GATCGGTCGAATGCGTGT	
*intI*2-LC2	TGCTTTTCCCACCCTTACC	*intI*2
*intI*2-LC3	GACGGCTACCCTCTGTTATCTC	
*intI*3-LC1	GCCACCACTTGTTTGAGGA	*intI*3
*intI*3-LC2	GGATGTCTGTGCCTGCTTG	

*intI*1-probe	ATTCCTGGCCGTGGTTCTGGGTTTT	*intI*1
*intI*2-probe	TGGATACTCGCAAACAAGTTATTTTTACGCTG	*intI*2
*intI*3-probe	CGCCACTCATTCGCCACCCA	*intI*3

**Table 2:** Comparison of carbapenem nonwild type *Klebsiella pneumoniae* strains with and without carbapenemase production.

		Carbapenemase producers (*n* = 15)	Carbapenemase nonproducers (*n* = 58)
Country	Russia	15	7
	Estonia	0	37
	Latvia	0	8
	Lithuania	0	6

Department	Intensive care	14	8
	Surgical	0	16
	Medical	1	31
	Outpatients	0	3

Material	Blood	1	1
	Pus/wound	2	11
	Urine	5	39
	Lower respiratory	7	6
	Upper respiratory	0	1

Genes detected (number of positive strains)	NDM	15	0
	IMP, VIM, GIM, KPC or OXA48	0	0
	CTX-M-1 group	13	50
	CTX-M-2 group	0	2
	CTX-M-8 or 9	0	0
	Integron 1	15	52
	Integron 2	0	1
	Integron 3	11^1^	25^1^

MIC values, mg/L (range/median)	Ertapenem	≥32^2^	0.125–≥32/6^2^
	Meropenem	4–≥32/12^3^	0.032–4/0.5^3^
	Imipenem	8–≥32/32^4^	0.094–4/0.5^4^

Resistance (numbers: *R* + *I*/tested strains)	Ciprofloxacin	15/15	49/51
	Gentamycin	15/15^5^	34/49^5^

^1^ *P* = 0.04; ^2,3,4^ *P* = 0.001; ^5^ *P* = 0.01.
